# A maChine and deep Learning Approach to predict pulmoNary hyperteNsIon in newbornS with congenital diaphragmatic Hernia (CLANNISH): Protocol for a retrospective study

**DOI:** 10.1371/journal.pone.0259724

**Published:** 2021-11-09

**Authors:** Ilaria Amodeo, Giorgio De Nunzio, Genny Raffaeli, Irene Borzani, Alice Griggio, Luana Conte, Francesco Macchini, Valentina Condò, Nicola Persico, Isabella Fabietti, Stefano Ghirardello, Maria Pierro, Benedetta Tafuri, Giuseppe Como, Donato Cascio, Mariarosa Colnaghi, Fabio Mosca, Giacomo Cavallaro

**Affiliations:** 1 NICU, Fondazione IRCCS Ca’ Granda Ospedale Maggiore Policlinico, Milan, Italy; 2 Department of Mathematics and Physics “E. De Giorgi”, Laboratory of Biomedical Physics and Environment, Università del Salento, Lecce, Italy; 3 Advanced Data Analysis in Medicine (ADAM), Laboratory of Interdisciplinary Research Applied to Medicine (DReAM), Università del Salento, Lecce, Italy; 4 Azienda Sanitaria Locale (ASL), Lecce, Italy; 5 Department of Clinical Sciences and Community Health, Università degli Studi di Milano, Milan, Italy; 6 Pediatric Radiology Unit, Fondazione IRCCS Ca’ Granda Ospedale Maggiore Policlinico, Milan, Italy; 7 Monza and Brianza Mother and Child Foundation, San Gerardo Hospital, Università degli Studi di Milano-Bicocca, Monza, Italy; 8 Department of Pediatric Surgery, Fondazione IRCCS Ca’ Granda Ospedale Maggiore Policlinico, Milan, Italy; 9 Department of Obstetrics and Gynecology, Fondazione IRCCS Ca’ Granda, Ospedale Maggiore Policlinico, Milan, Italy; 10 NICU, Bufalini Hospital, Azienda Unità Sanitaria Locale della Romagna, Cesena, Italy; 11 Department of Physics and Chemistry, Università degli Studi di Palermo, Palermo, Italy; Medicina Fetal Mexico, MEXICO

## Abstract

**Introduction:**

Outcome predictions of patients with congenital diaphragmatic hernia (CDH) still have some limitations in the prenatal estimate of postnatal pulmonary hypertension (PH). We propose applying Machine Learning (ML), and Deep Learning (DL) approaches to fetuses and newborns with CDH to develop forecasting models in prenatal epoch, based on the integrated analysis of clinical data, to provide neonatal PH as the first outcome and, possibly: favorable response to fetal endoscopic tracheal occlusion (FETO), need for Extracorporeal Membrane Oxygenation (ECMO), survival to ECMO, and death. Moreover, we plan to produce a (semi)automatic fetus lung segmentation system in Magnetic Resonance Imaging (MRI), which will be useful during project implementation but will also be an important tool itself to standardize lung volume measures for CDH fetuses.

**Methods and analytics:**

Patients with isolated CDH from singleton pregnancies will be enrolled, whose prenatal checks were performed at the Fetal Surgery Unit of the Fondazione IRCCS Ca’ Granda Ospedale Maggiore Policlinico (Milan, Italy) from the 30^th^ week of gestation. A retrospective data collection of clinical and radiological variables from newborns’ and mothers’ clinical records will be performed for eligible patients born between 01/01/2012 and 31/12/2020. The native sequences from fetal magnetic resonance imaging (MRI) will be collected. Data from different sources will be integrated and analyzed using ML and DL, and forecasting algorithms will be developed for each outcome. Methods of data augmentation and dimensionality reduction (feature selection and extraction) will be employed to increase sample size and avoid overfitting. A software system for automatic fetal lung volume segmentation in MRI based on the DL 3D U-NET approach will also be developed.

**Ethics and dissemination:**

This retrospective study received approval from the local ethics committee (Milan Area 2, Italy). The development of predictive models in CDH outcomes will provide a key contribution in disease prediction, early targeted interventions, and personalized management, with an overall improvement in care quality, resource allocation, healthcare, and family savings. Our findings will be validated in a future prospective multicenter cohort study.

**Registration:**

The study was registered at ClinicalTrials.gov with the identifier NCT04609163.

## Introduction

Congenital Diaphragmatic Hernia (CDH) is a rare congenital malformation that affects 1–4 newborns per 10.000 live births, characterized by a diaphragmatic defect, which allows the herniation of the abdominal organs into the thorax [1, 2].

CDH distinctive features are pulmonary hypoplasia and postnatal pulmonary hypertension (PH). The decreased bronchial branching and reduced gas-exchange surface area are invariably associated with impaired vascular development, which is characterized by reduced extension and remodeling of the vascular network and altered vasoreactivity [1, 3–8]. However, the pathogenesis of PH has not been fully clarified yet [9–18]. In addition, the degree of postnatal respiratory and cardiovascular compromise are key determinants of prognosis [19, 20].

Neonatal survival is approximately 70% but varies from over 90% in mild CDH to limited chances of survival (less than 10%) in extreme forms depending on several factors, such as defect side and size, associated anomalies, gestational age at birth, and treatment [21–24]. In addition, survivors may have chronic lung disease, persistent PH, feeding and growth problems, gastroesophageal reflux, hearing loss, neurocognitive delay, thoracic deformations, and hernia recurrence [25].

Early detection of fetuses with limited chances of survival allows corrective prenatal intervention. Indeed, fetal endoscopic tracheal occlusion (FETO) performed at 27–29 weeks with an endovascular detachable latex balloon (Goldbal 2) counteracts the herniation of abdominal organs and promotes lung development through the accumulation of lung fluid, eventually improving survival. Balloon removal is usually scheduled at 34 weeks [24, 26]. Results from a multicenter randomized clinical trial investigating the survival increase after the procedure in left-sided severe and moderate CDH have recently been published (www.totaltrial.eu) [27, 28]. It can also be clinically offered to severe right-sided CDH [24].

After birth, treatment strategies include mechanical ventilation, drug therapies, and surgical correction to restore normal anatomy [25, 29, 30]. However, in case of failure of conventional therapies, Extracorporeal Membrane Oxygenation (ECMO) may be required [31, 32]. Indeed, CDH represents the main neonatal ECMO indication, with an overall survival rate of about 50% of the cases [24, 31, 32]. Besides, ECMO is an invasive and high-risk procedure, possibly associated with acute complications and long-term morbidities [32–34].

Efforts have been made to identify prenatal and postnatal indicators to improve outcome prediction and individualized management. The combined evaluation of lung size, liver position, and defect side through prenatal imaging is conventionally accepted to stratify CDH fetuses in different groups, correlating with perinatal mortality and long-term morbidity [22, 35]. The calculation of the observed/expected lung-to-head ratio (O/E LHR) with obstetric ultrasound (US) and the observed/expected total fetal lung volume (O/E TFLV) with fetal magnetic resonance imaging (MRI) are used to assess lung hypoplasia and subsequently predict neonatal outcomes [36]. The size of the defect and hernia sacs may serve as additional predictive tools [36–40]. However, the prognostic accuracy of these parameters has some limitations, especially for postnatal PH [35]. In addition, associated malformations and genetic anomalies have to be ruled out, as they influence the prognosis [24, 41].

Although fetal lung volume reflects lung development, it represents only an approximation of the vascular network’s extent and does not consider all the physiological variables influencing vascular resistance [35, 42]. Ideally, we should predict pulmonary hypertension independently from lung volumetry since PH itself correlates with mortality and long-term morbidity [43]. However, techniques available for directly measuring lung vascularization are not easily reproducible, and their predictive values remain unclear [44–51]. Moreover, in addition to increased vascular resistance, decreased pulmonary blood flow may be affected by vessel distortion caused by visceral herniation itself, which makes the measurement of prognostic parameters less accurate [42]. Finally, the hemodynamic changes occurring during the transition from fetal to neonatal life make the exact prediction of postnatal pulmonary vascular resistance and perfusion trends challenging [43].

In light of these considerations, the prenatal prediction of postnatal PH still represents the weakest point of the prognostic assessment of CDH [36, 42, 43].

Recently, methodologies based on artificial intelligence (AI) have been developed to support the analysis of medical data, in particular the traditional Machine Learning (ML) approach and its modern extension, Deep Learning (DL) [52]. In different but integrated ways, both methods explore the possibility of building forecasting algorithms, starting from acquiring relevant clinical data and using them to predict a specific outcome, anticipate adverse events, guide interventions, and improve the overall quality of care [52, 53].

The application of these novel technologies, although still limited, is also spreading in the neonatal field [54–60]. Nevertheless, to the best of our knowledge, they have not been successfully applied to CDH newborns yet, and a specific prenatal prediction of the disease outcome still lacks [61].

Our hypothesis is that combined ML and DL systems in prenatal epoch could allow forecasting algorithms for outcome prediction in newborns with CDH.

## Methods and analysis

### Study design

A retrospective data collection and study will be performed at Fondazione IRCCS Ca’ Granda Ospedale Maggiore Policlinico, Milan, Italy, involving the Fetal Surgery Center, Pediatric Radiology Service, Pediatric Surgery Unit, and Neonatal Intensive Care Unit (NICU). At the same time, ML and DL data analyses, and the development of prototype predictive models and segmentation algorithms, will be started at the Department of Mathematics and Physics of the Università del Salento, Lecce, Italy, and at the Department of Physics and Chemistry of the Università degli Studi di Palermo, Palermo, Italy.

**Fig 1:**
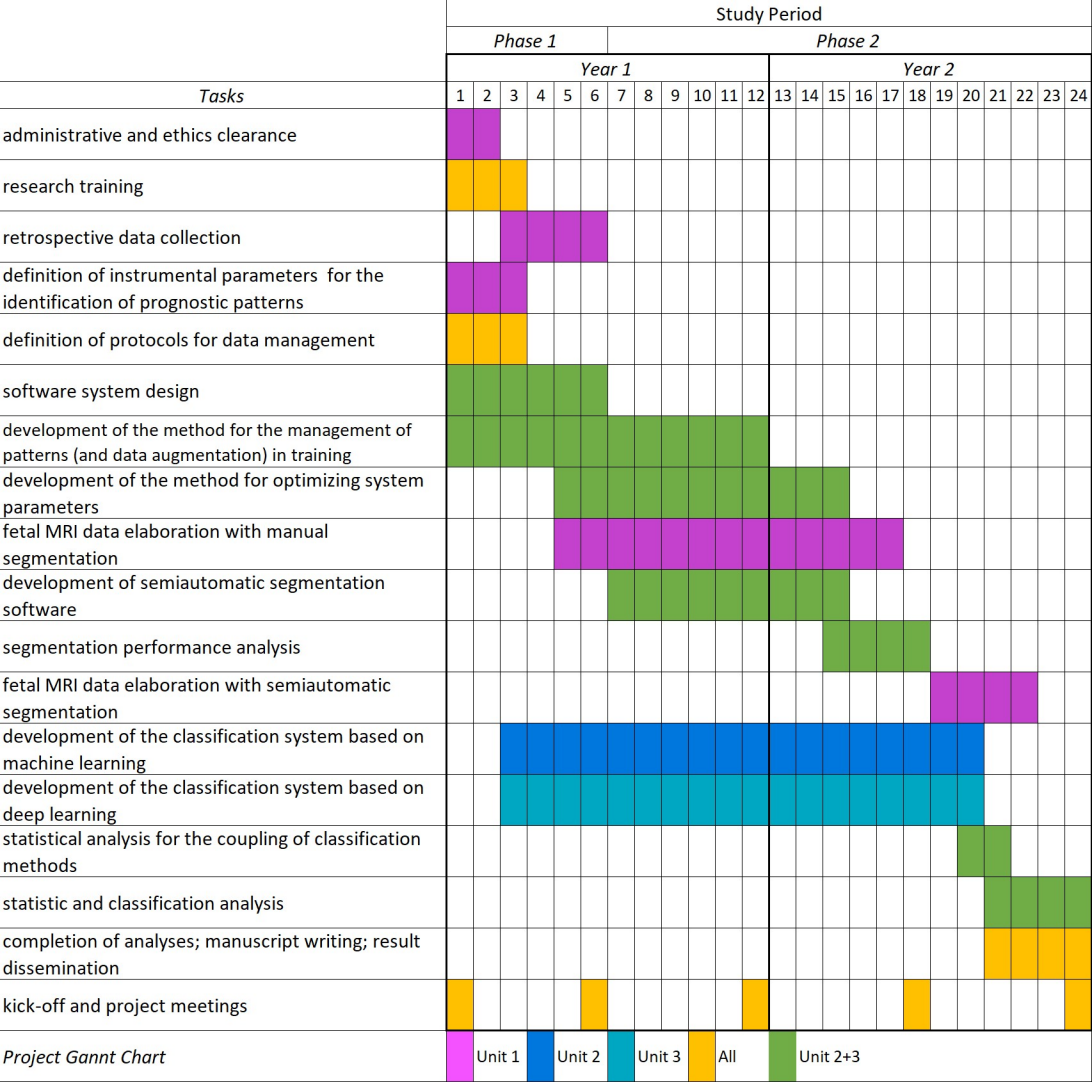
Standard protocol items. Standardized Protocol ItemsRecommendations for Observational Retrospective Study (SPIROS) flow diagram: Schedule for administrative and ethics clearance; research training; retrospective data collection; definition of instrumental parameters for the identification of prognostic patterns; definition of protocols for data management; software system design development of the method for the management of patterns (and data augmentation) in training; development of the method for optimizing system parameters; fetal MRI data elaboration with manual segmentation; development of semiautomatic segmentation software; segmentation performance analysis; fetal MRI data elaboration with semiautomatic segmentation; development of the classification system based on machine learning; development of the classification system based on deep learning; statistical analysis for the coupling of classification methods; statistic and classification analysis; completion of analyses; manuscript writing; result dissemination; kick-off and project meetings. Unit 1: Milan; Unit 2: Lecce; Unit 3: Palermo.

We identified two main project phases. During the first six months, the first phase includes all administrative and ethics clearance, the research team’s training, and the collection of retrospective data collection. Moreover, the definition of instrumental parameters for identifying prognostic patterns, the definition of protocols for data management, and the software system design. The second phase involves the machine and deep learning analysis and will end 18 months later (Fig 1). The prototype ML- and DL-based predictive prognostic models will be obtained and integrated into a mixed ML-DL system to be then applied to the prospective data. The segmentation software prototype will be used as soon as it is available. The software tools will be tested and optimized during the project’s progress (Fig 1).

### Patient involvement

Parents of newborns with CDH were not involved in the design, or conduct, or reporting, of our research, but they will be involved in our dissemination plans.

### Study population and sample size

Patients with CDH born between 01/01/2012 and 31/12/2020 will be considered for the study. During the study period, the number of eligible patients will be about 80 subjects. Calculating a 30% drop-out due to exclusion criteria, we will enroll about 56 subjects with CDH.

**Fig 2:**
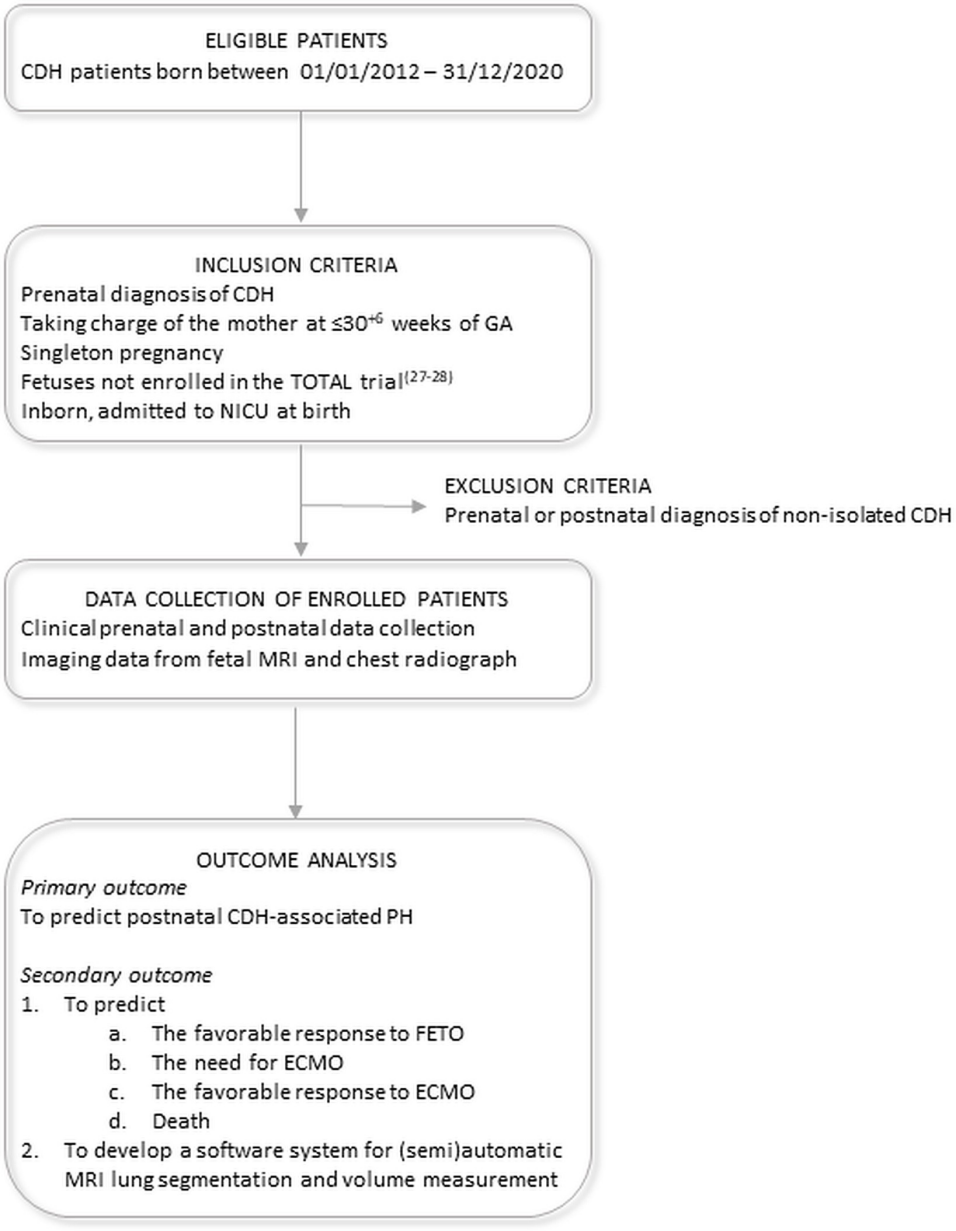
Study flow chart. CDH: Congenital diaphragmatic hernia; GA: Gestational age; NICU: Neonatal intensive care unit; MRI: Magnetic resonance imaging; PH: Pulmonary hypertension; FETO: Fetal endoscopic tracheal occlusion; ECMO: Extracorporeal membrane oxygenation.

Enrollment will be performed according to the following criteria (Fig 2):

Inclusion criteria (all of these):

- Prenatal diagnosis of CDH;
- Prenatal taking charge of the mother with CDH fetus at a gestational age below or equal to 30^+6^ weeks at our Fetal Surgery Center;
- Singleton pregnancy;
- Fetuses not enrolled in the TOTAL trial (https://www.totaltrial.eu/);
- Inborn patients admitted to the NICU at birth;

Exclusion criteria:

- Prenatal or postnatal diagnosis of non-isolated CDH, thus associated with other malformations or genetic anomalies.

### Data collection

Clinical and instrumental data regarding the prenatal history and the medical and surgical postnatal course of each patient, based on mothers’ and newborns’ medical records, will be collected (Astraia, Astraia Software GmbH; NeoCare, GPI SpA) (S1 Table).

Prenatal ultrasound performed between 25^+0^ and 30^+6^ weeks of gestation (before FETO procedure, in case of prenatal treatment) for the following data will be considered: estimated fetal weight; amniotic fluid; defect side; herniated organs; O/E LHR% (tracing method); grading of hernia severity; doppler parameters (umbilical artery pulsatility index, pulmonary artery pulsatility index, pulmonary artery peak systolic velocity, pulmonary artery peak early diastolic reverse flow) [22, 62–65]. Gestational age at diagnosis, details about FETO procedure, and pregnancy course will also be recorded. In particular, a favorable response to FETO in terms of survival will be considered.

Regarding the neonatal course, PH will be the main focus. In the setting of CDH, PH is defined as elevated pulmonary vascular resistance relative to systemic blood pressure, based on echocardiographic and clinical parameters [66]. Systolic pulmonary artery pressure from tricuspid valve regurgitation, mean pulmonary artery pressure from pulmonary valve regurgitation, pulmonary artery flow, characteristics of the interventricular sept, presence, and characteristics of shunts, will be obtained from the systematic review of cardiac US performed bedside during NICU stay. In particular, we will consider: the earliest echocardiogram after NICU admission performed within the first day of life (T0), the pre- (T1) and postoperative (T2) assessment performed closest to CDH repair, and echocardiogram performed one week after surgery (T3) [67]. Physiological parameters such as systemic arterial pressure, heart rate, oxygen pre-and post-ductal saturation will be obtained from the electronic monitoring systems records throughout the hospitalization and matched with the echocardiographic assessment to define the presence of CDH-associated PH. Patients with systemic or suprasystemic pulmonary artery pressure will be categorized as having CDH-associated PH.

From each newborn’s electronic medical record, data regarding the following treatments will be extracted: mechanical ventilation, oxygen supplementation, pulmonary vasodilators, vasoactive and inotropic support, antibiotics, blood product transfusions.

The vasoactive inotropic score (VIS) will be calculated hourly, and the maximum VIS will be recorded at each of the four time points mentioned above [68, 69].

Trends of laboratory parameters will also be collected, like complete blood count and differential, hemoglobin, C-reactive protein, bilirubin.

The surgical course, day of the intervention, type of surgical repair, prosthetic patch use, intra- or postoperative complications will be noted. In addition, all ECMO cases and deaths during the hospital stay will be reported. In particular, a favorable response to ECMO in terms of survival to procedure and/or discharge will be considered.

Multiple checks will be performed to assess data quality, integrity, and accuracy. Any incorrect or inconsistent data will be reviewed and verified through the revision of medical records.

### Data extraction

Data used for the study will be extracted from NeoCare electronic medical record (GPI SpA). The relational database will provide access to data points related to each other. The relational database will be based on the relational model, and the data will be stored in related tables. Starting from the initial dataset of the patients enrolled for the study using Sequential Query Language (SQL), data of interest for the analysis for each patient will be extracted. Two different types of data will be extracted:

1. structured data per patient, for which there is only one data for each patient;
2. time-series data for which multiple data per patient will be extracted, associated with recording date and time.

### Radiological parameters

**Fig 3:**
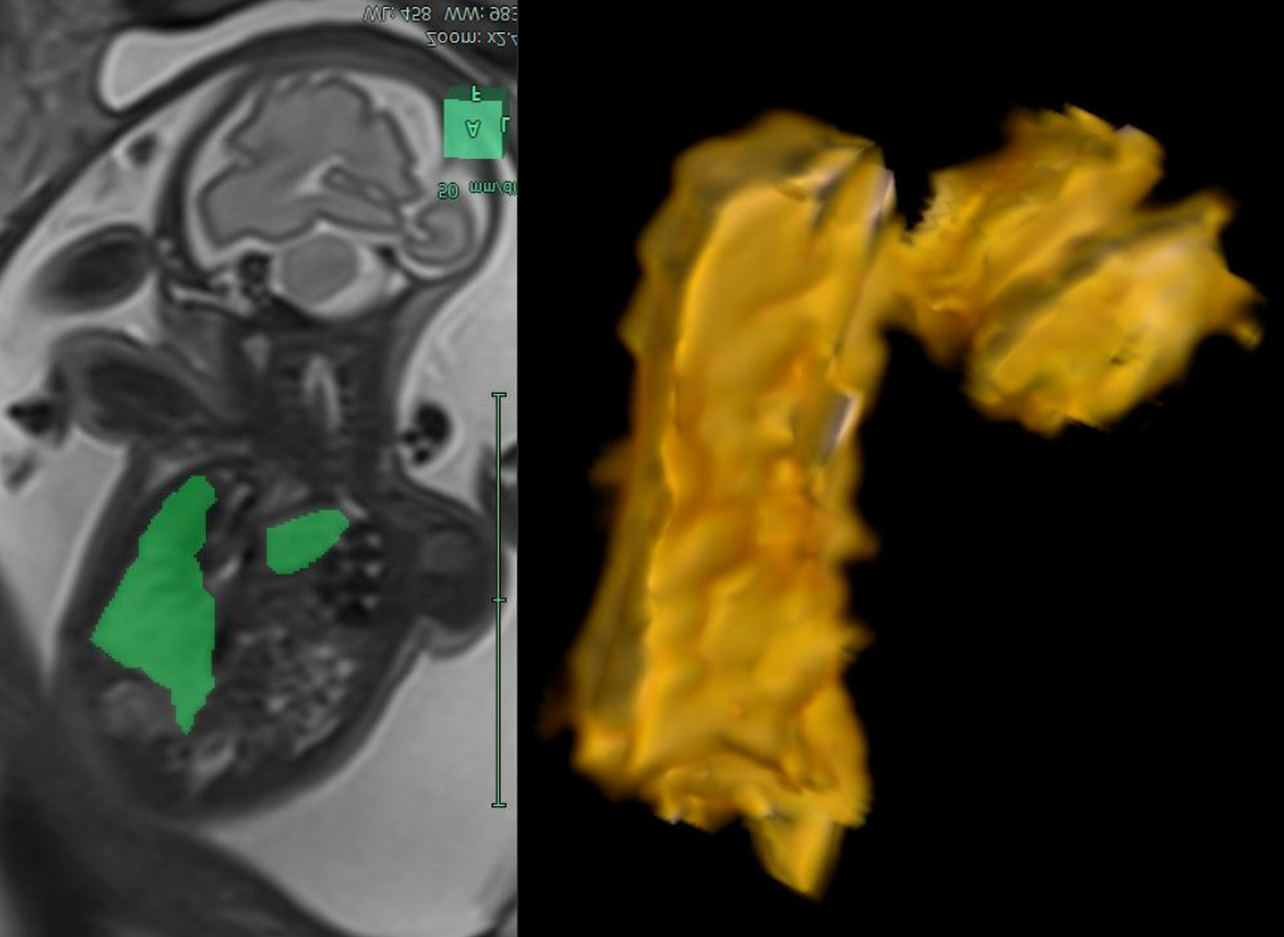
Total fetal lung volume assessment. MRI segmentation and 3D reconstruction of the fetal lung with the calculation of the total fetal lung volume on T2-sequences.

**Fig 4:**
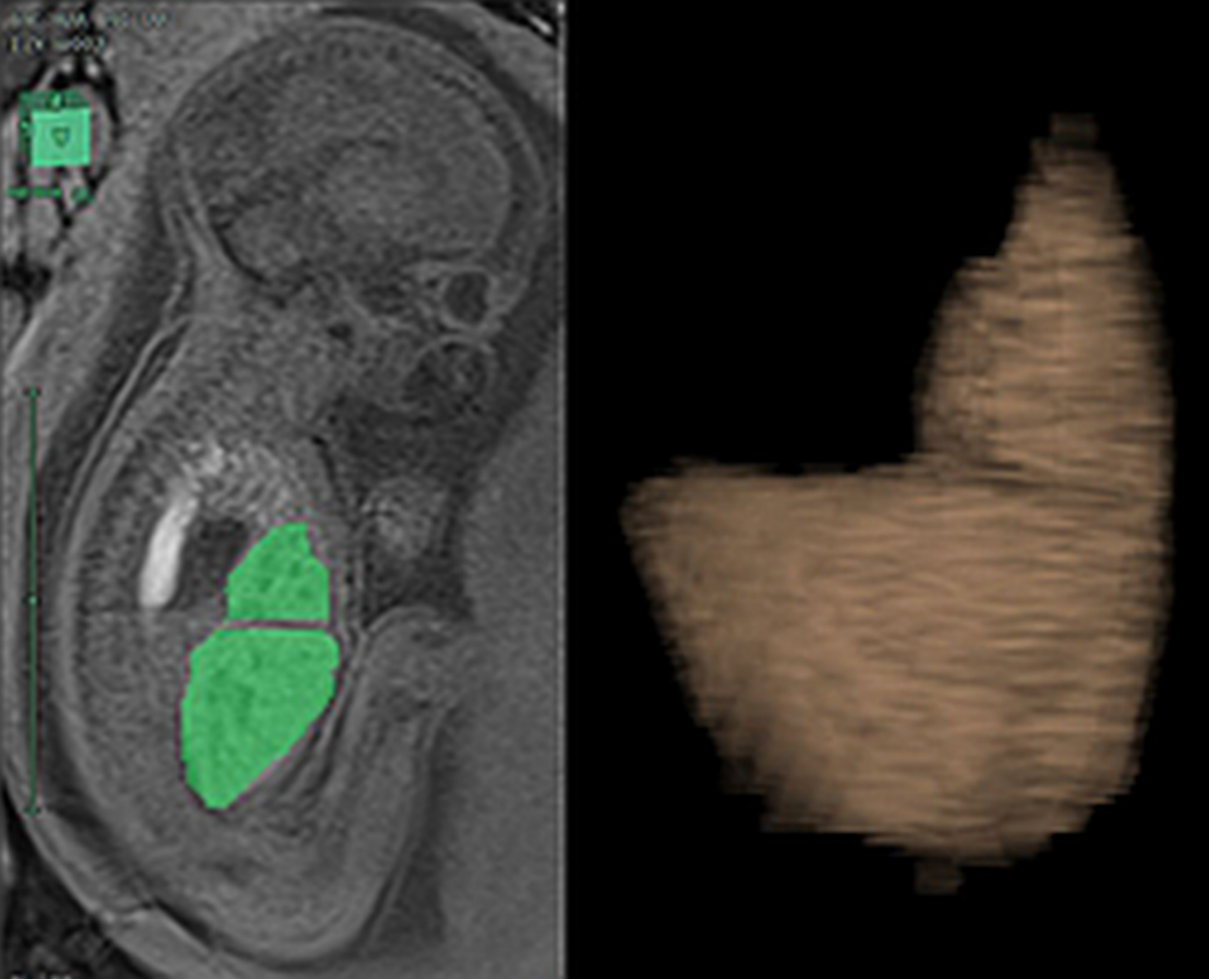
Liver volume assessment. MRI segmentation and 3D reconstruction of the fetal liver with the calculation of the herniated liver on T2-sequences.

**Fig 5:**
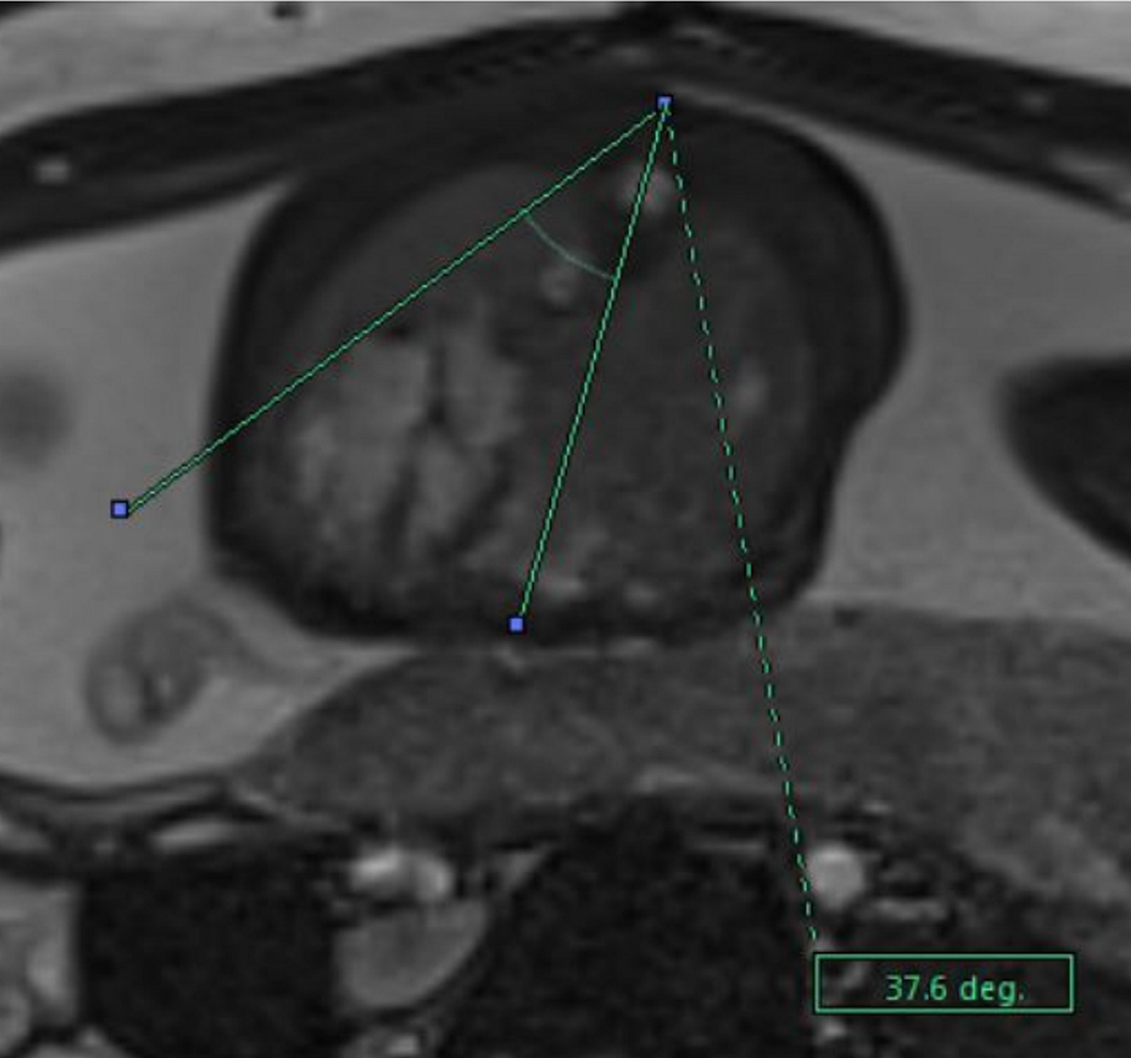
Mediastinal shift angle. MRI calculation of the mediastinal shift angle (MSA) on axial TrueFisp-sequences.

**Fig 6:**
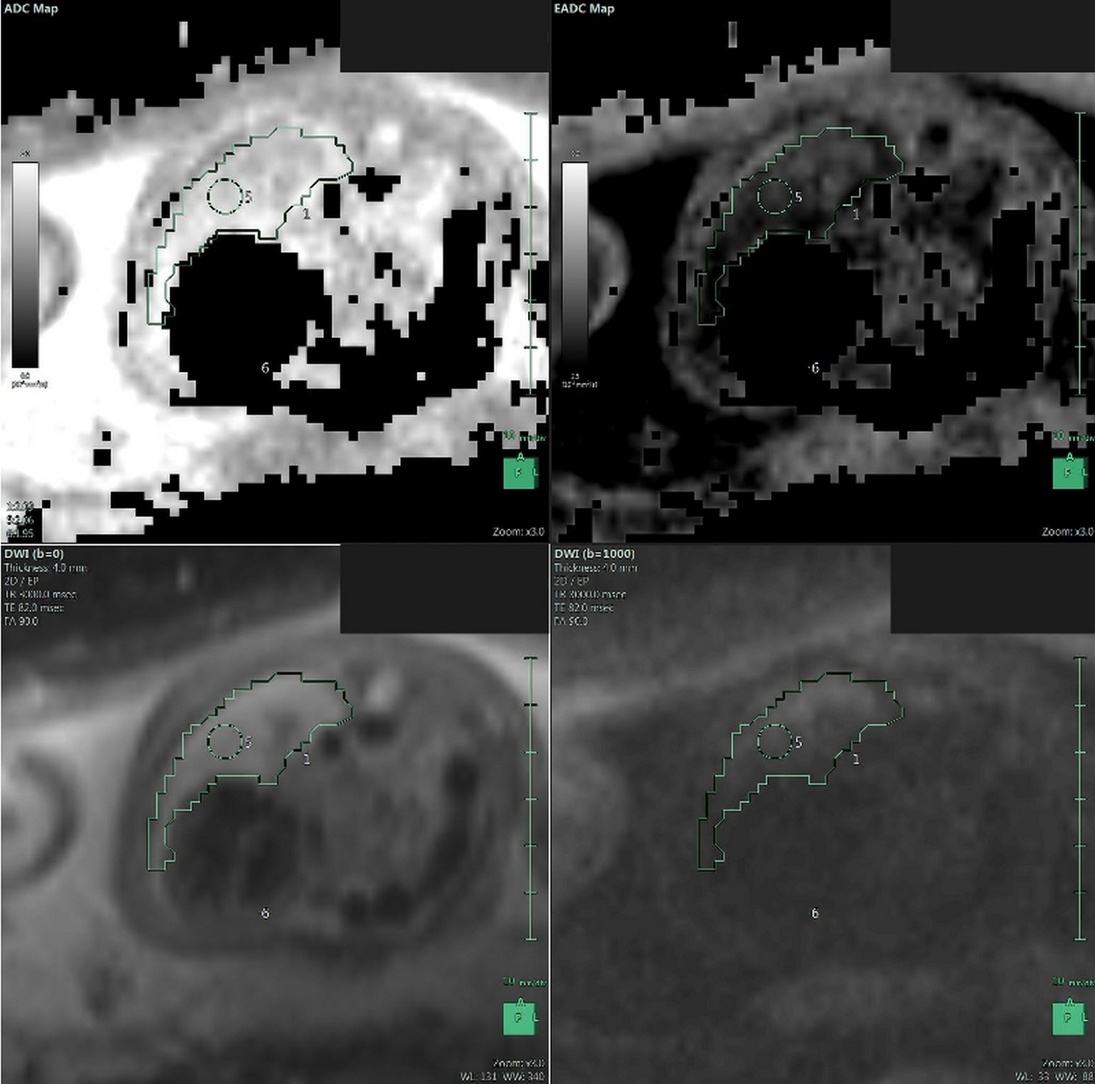
Apparent diffusion coefficient. MRI calculation of the apparent diffusion coefficient (ADC) on diffusion-weighted sequences.

The software imaging in use is Synapse PACS and Synapse 3D (FUJIFILM Medical Systems USA, Inc.). All fetal MRIs performed soon after the US diagnosis of CDH and before fetal intervention in those fetuses eligible for the procedure (25^+0^–35^+6^ weeks) will be considered. In addition, lung volumes, liver volume, mediastinal shift angle (MSA), and apparent diffusion coefficient (ADC) for each patient will be calculated (Figs 3–6) [69–72].

Lung volumes will be calculated on the T2 HASTE sequences, selecting the plane corresponding to the best image quality, covering the whole thorax on a single acquisition without motion-induced artifacts. On each section, left and right lung areas will be independently determined by drawing freehand regions of interest (ROIs). The main vessels of the pulmonary hila and mediastinal structures will be excluded. The areas will be automatically added, multiplied by the sum of slice thickness and intergap by the software, to obtain the entire volume of each lung. Left and right volumes will be added to obtain the total fetal lung volume (TFLV). The TFLV will be expressed as a percentage of the mean standard value expected for gestational age (O/E TFLV%), as determined by Rypens et al. [73].

The liver volume will be calculated with the same technique. The proportion between the liver volume above the diaphragmatic level and the total volume will be calculated and expressed as a percentage of liver herniation (%LH) [74, 75].

The ADC will be calculated using a freehand ROI on each lung, considering the median, minimum, and maximum values [72].

The mediastinal shift angle (MSA) will be measured on an axial True-Fisp image at the level of the four-chamber view of the fetal heart. A sagittal midline landmark line will be drawn from the posterior face of the vertebral body to the mid of the posterior surface of the sternum, dividing the fetal thorax into two symmetric parts. A second landmark line will be traced from the same vertebral point, representing the angle vertex, tangentially to the external wall of the right atrium [69, 71, 76, 77]. The software will automatically calculate the corresponding MSA.

**Fig 7:**
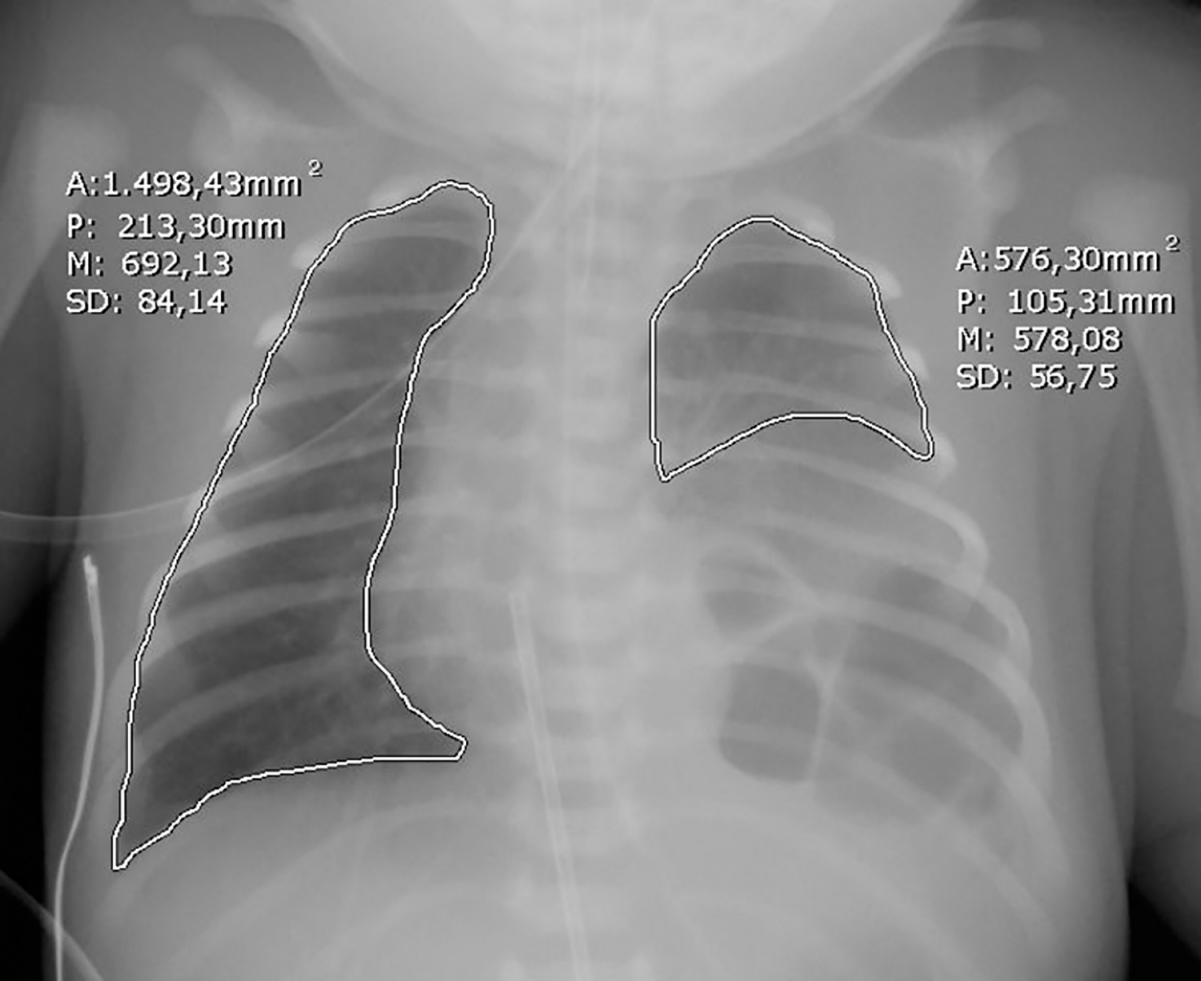
Chest radiographic pulmonary area. Calculation of the radiographic pulmonary area on neonatal chest x-ray.

Finally, the radiographic pulmonary area will be calculated on a digital chest x-ray performed within 24 hours after birth by tracing the perimeter of the lung outlined by the rib cage and the diaphragm, excluding the mediastinal structures and the herniated organs (Fig 7) [67, 78, 79].

### Outcome

*Primary outcome*. To build an AI system able to identify prenatally patients who will develop CDH-associated PH at T0, T1, T2, T3.

*Secondary outcome*.

1. To build models able to predict:
  - the favorable response to FETO: survival to NICU discharge in patients undergoing the procedure;
  - the need for ECMO;
  - the favorable response to ECMO treatment: survival to ECMO and survival to NICU discharge in patients undergoing the procedure;
  - death.
2. To develop a software system for (semi)automatic fetal lung segmentation and volume measurement in MRI.

### Analysis

Data will be analyzed using ML, and DL approaches. This computational analysis belongs to the domain of AI and supervised pattern recognition. ML is a traditional technique that provides algorithms that parse data, learn from data without being explicitly programmed, and then apply what they have learned to make informed decisions. The typical ML pipeline consists of multiple sequential steps that perform data acquisition and preprocessing, calculation from the data of a usually large number of variables (also called attributes or features, both domain-specific and “agnostic”), complexity reduction, model training and validation, deployment [80]. In the training phase, the ML model learns the possible relationship between the features and a target variable (the outcome of interest), and in principle, generalizes, becoming able to give the correct value of the target variable even for unseen samples.

DL methods are a newer application of ML. While in classical ML techniques, the most effective features need to be identified by a domain expert and/or by laborious trials, the biggest advantage of DL algorithms is that they try to learn high-level features from data in a direct and incremental manner. This eliminates the need of domain expertise and hard-core feature extraction.

The purpose of ML and DL classification methods will be fetus stratification, i.e., to build models in which patients are classified according to the different outcomes of interest (primarily the possible development of PH, but also response to FETO, need and response to ECMO, death).

Computing environments will be Python and Matlab/Octave. The computational system will be based on the combined and multivariate use of features from clinical and instrumental data acquired in the pre-and postnatal period. For the analysis of MRI images, we will create ad hoc computing tools that will perform the following steps: import of the native image, (semi)automatic contouring and segmentation of the volumes of interest, calculation of semantic and agnostic feature descriptors, classification by ML and DL methods, using commercial software or preferably open-source libraries.

After contouring/segmentation, feature descriptors for supervised pattern recognition by ML techniques will be calculated in the volumes of interest (particularly the fetus lung and liver). Initially, expert radiologists will manually execute segmentation, but in progress, we will also develop a (semi)automatic segmentation software useful to supplement the radiologists’ work.

For the segmentation phase, a 3D U-Net will be implemented [81]. The 3D U-Net has recently been proposed and has been widely used for volumetric segmentation in medical images due to its outstanding performance. It is an extended version of the previously proposed 2D U-Net. It must be said that fully convolutional neural networks (CNNs) like U-Net have been the dominant approach in automatic medical imaging segmentation [82, 83]. This architecture has been designed to work with a very small number of training images, and this allows a high-performance application to many medical imaging problems in which it is easy to have a small number of data. It consists of two architectural parts: contracting path and expanding path. To learn and use local information, high-resolution 3D features in the contracting path are concatenated with upsampled versions of global low-resolution 3D features in the expanding path. Through this concatenation, the network learns to use both high-resolution local features and low-resolution global features. The network will be trained and optimized for fetal lung volume segmentation in MRI.

To cope with the small sample size issue, we plan to implement data augmentation techniques [84]. Data augmentation increases the amount of available data by adding slightly modified copies of existing data or synthetic data derived from existing ones to the dataset. Usually, data augmentation makes the models more robust and reduces overfitting. Several methods will be used, such as: exporting the images of each patient from the PACS with different spatial orientations, rototranslations of the images, slightly modifying the computed feature descriptors. Through data augmentation, the sample size—although originally limited by the small number of patients–will grow to the advantage of predictive model training.

In the ML approach, the calculated features will comprise specific clinical variables (as described in the “Data Collection” and “Radiological Parameters” sections), further semantic features computed from the diagnostic images, and Radiomics agnostic features [85]. As the most evident effect of herniation is the distortion of organ forms, quantitative shape analysis will be used to precisely locate and measure morphological changes.

Once a clinical and imaging feature vector is defined, the result will necessarily be a dataset with many variables compared to the number of cases analyzed. This may lead to a peculiar phenomenon known as the “curse of dimensionality”, in which a large number of parameters (features) gives origin to poor classification quality because, as it is well known, the number of samples needed to estimate an arbitrary function with a high level of accuracy grows exponentially with the number of variables [86]. Therefore, a dimensionality reduction phase will be performed in order to eliminate correlated or irrelevant data and to preserve more discriminant variables, overall reducing dimensionality [87]. Methods such as the Principal Component Analysis (PCA), Independent Component Analysis (ICA), Fisher Linear Discriminant Analysis (FLDA), and various feature selection techniques will be used. PCA, ICA, and FLDA are known as extraction methods and allow the computation of a reduced-size set of novel features starting from a large initial set. On the other hand, feature selection reduces dimensionality by choosing a set of “best” features, i.e., the ones which maximize classification quality, discarding redundant or useless ones. An advantage of feature selection, besides dimensionality reduction, is also that by identifying the best features it gets human-understandable insights, to the advantage of the so-called ML “explainability” (i.e., reducing the “black box” character that ML applications often have) [88].

**Fig 8:**
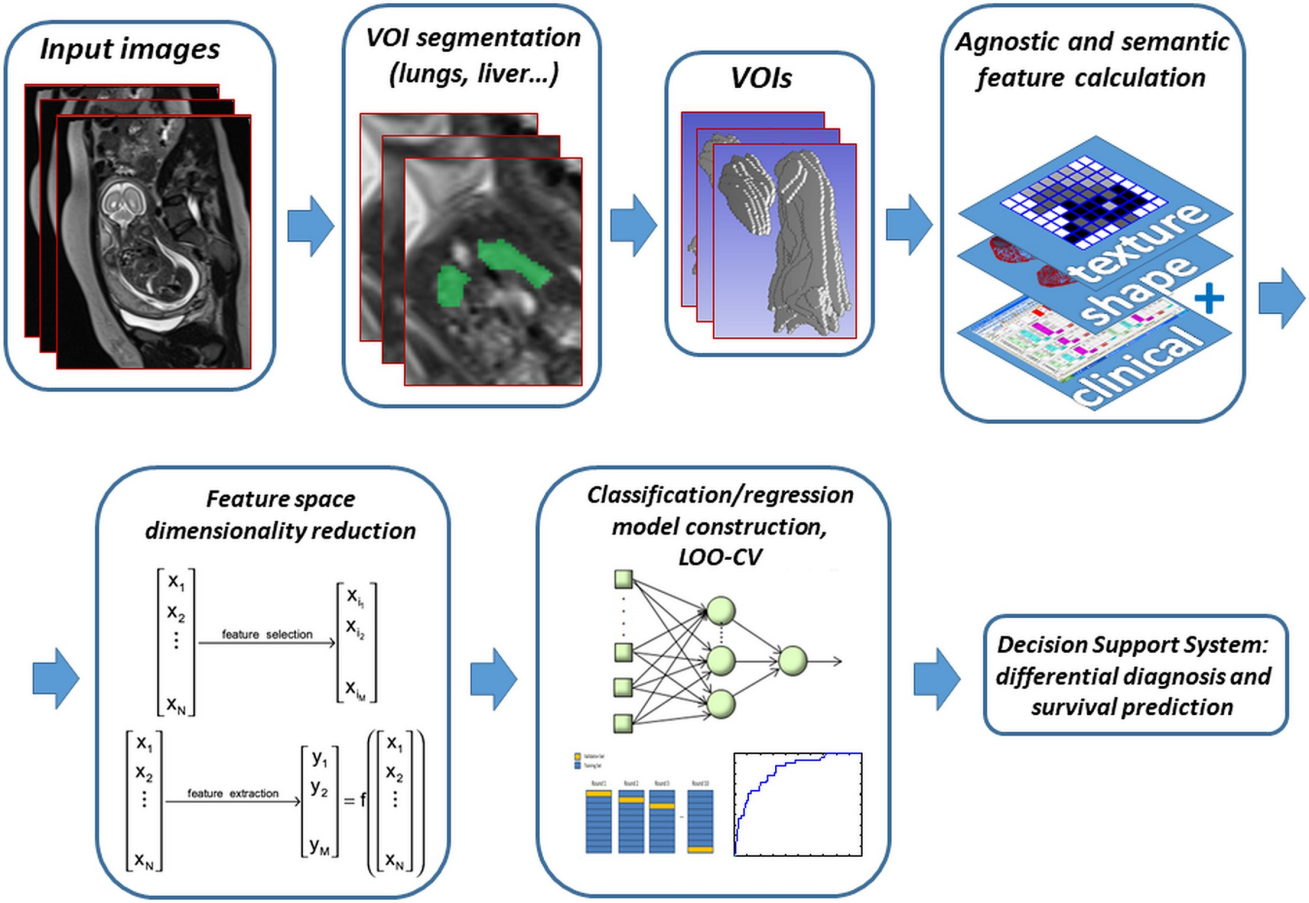
Machine learning pipeline. The flowchart illustrates the radiomics workflow starting from multimodal image acquisition. After manual or (semi)automatic segmentation/contouring of the volumes of interest (such as fetus lungs and liver), feature descriptors are calculated from VOI shape and texture. Together with a choice of prenatal clinical parameters, the obtained feature vectors are labeled with relevant output variables (e.g., presence of postnatal PH, or need for ECMO…) and, after dimensionality reduction performed to get rid of redundant and useless descriptors, enter the supervised classification/regression model-construction step, here represented by one of the possible choices, i.e., a multi-layer perceptron artificial neural network. Because of the relatively low number of samples, model training and validation will be obtained by leave-one-out cross-validation (LOO-CV), and various methods, such as the ROC curve, will quantify model quality. The trained and validated ML model will be the pipeline output to be employed in the Decision Support System.

Classification of feature vectors will be performed through conventional feed-forward Artificial Neural Networks, Support Vector Machines (SVM), and other classification systems [80]. In addition, the usage of Ensemble Learning systems is planned, such as Random Forests. In order to make maximum use of the examples available, training/validation will be performed with a Leave One Patient Out Cross Validation scheme (LOPO-CV). The performance of the trained system will be measured by the Receiver Operating Characteristic (ROC) curve and the sensitivity and specificity of the system (Fig 8).

**Fig 9:**
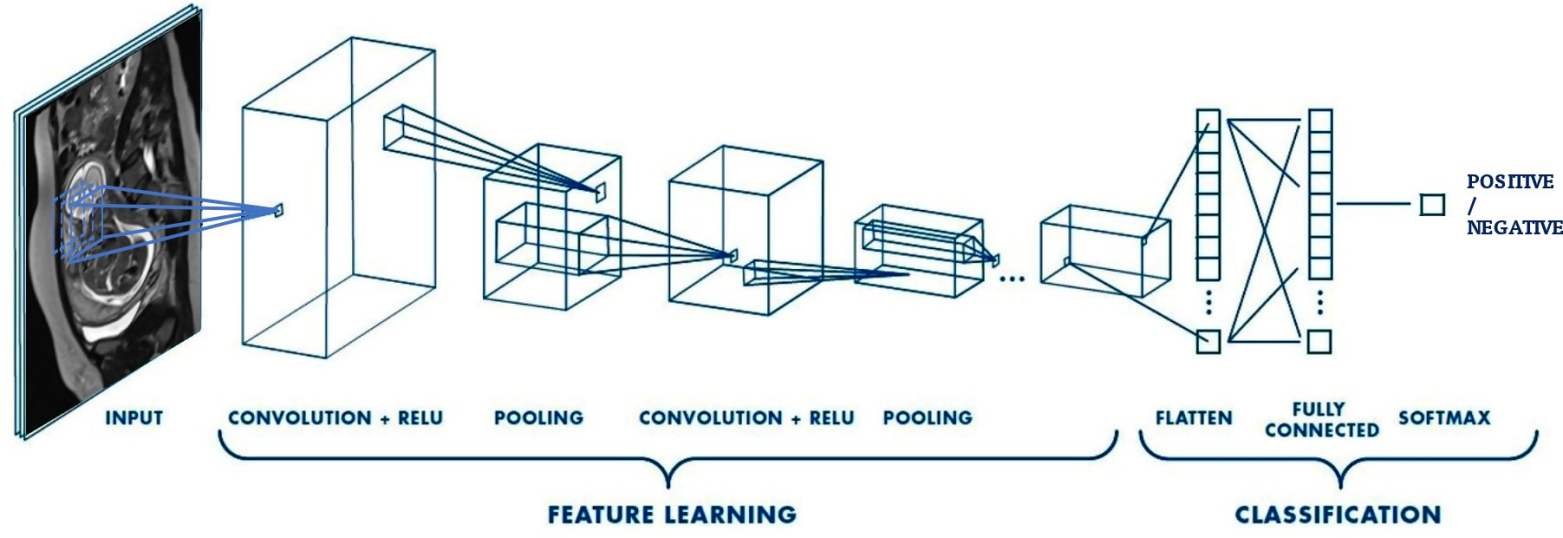
Schematic of the architecture of a convolutional neural network (CNN). A CNN is composed of several kinds of layers, namely the convolution layers and the pooling layers. One of the most significant differences between deep networks and other ML algorithms is the use of ReLU as a transfer function to make the algorithm faster. Then, the outputs generated by the previous levels are "flattened" to transform them into a single vector that can be used as an input for the next level. The fully connected layer applies weights to the input generated by the feature analysis to predict an accurate label. Finally, the fully connected output layer and softmax produce the final outputs in order to determine the class to associate with the image.

The classification effectiveness of DL will also be evaluated. In this project, four of the best-known pre-trained convolutional neural networks (CNNs) will be tested (AlexNet, SqueezeNet, ResNet18, GoogLeNet) (Fig 9) [89]. The fine-tuning training method will be implemented. In particular, different training modalities will be analyzed: three levels of freezing weights and scratch. Furthermore, the CNNs will be used as feature extractors; in this mode, the CNNs will be coupled to linear Support Vector Machine (SVM) classifiers. The main characteristic of SVM classification is their simplicity in terms of parameters, which allows them to face complex classification problems in which, as in our case, a large number of input features are present.

## Discussion

In the era of precision medicine research, integrating a wide variety of data from different sources is of key importance for the complete take charge of the patients [90]. The proposed retrospective project will be the first to explore the application of AI methods to CDH. Due to the complexity and heterogeneity of CDH patients, this disorder is well suited to an integrated, ML- and DL-based analysis of the multitude of clinical, instrumental, and imaging data deriving from multiple sources.

The use of AI methods will generate a unique advancement in managing fetal/neonatal patients with CDH. This innovative approach will maximize data collection from a rare clinical entity and allow the development of forecasting algorithms to predict neonates’ outcomes with CDH prenatally. The early detection of clinical and radiological prognostic factors will provide a peculiar opportunity to implement risk stratification, which is key for proper and timely prenatal counseling and guide resource-intensive procedures such as fetal surgery and neonatal ECMO support. Eventually, the selection of the right candidate for the right procedure in a timely manner will have a huge impact in terms of patient outcomes and resource allocation. Identifying the relationship between clinical-radiologic variables with patients’ outcomes will further clarify the main determinants of CDH-associated PH and better understand PH pathophysiology.

Moreover, imaging has increased its role in modern medicine over time, concerning the response to treatment, assessment of toxicity, and more recently in predicting results that allow for guiding therapeutic choices. Although medical imaging is rapidly growing, bias is still linked to human interpretation of the exam [91]. The segmentation task is fundamental for organ volume and shape assessment, but commonly used medical imaging software does not generally provide the physician with specific segmentation options; thus, contouring work is manual. Manual segmentation is a time-consuming process that tends to be operator-dependent and prone to errors, making volume and shape measurements made by different physicians often challenging to compare.

The definition of an automatic, or at least a semiautomatic, contouring and segmentation software tool specifically designed for the fetal lung would be relevant in clinical practice for fetal lung volume assessment, with obvious impact for healthcare in terms of standardization upgrade and simplification of the diagnostic process. As it will be the first software specifically created for fetal lung assessment, this innovative tool will improve data collection accuracy and create solid AI algorithms. This will optimize medical practice and resources, improve quality of care and outcomes, and benefit patients, families, healthcare professionals, and the public health system. If project evolution is favorable, we shall extend the segmentation software to liver contouring.

The multidisciplinary approach of this study requires the collaboration of experts and research units from different fields. Indeed, it gathers mathematicians, physicists, and clinical perinatal physicians and scientists, with different backgrounds and specific competencies, all equally important for the purpose of the study.

In light of these considerations, the present retrospective study could represent the first step of a possible new research line regarding patients with CDH. Indeed, from a research perspective, we intend to consolidate our results in a prospective, multicenter cohort study based on a larger CDH population. Shortly, we also intend to establish a Fetal Surgery/Neonatal Network supported by innovative software tools to enable data-driven decisions.

Our retrospective study is the first to explore the application of AI methods to CDH. While ML and DL fall under the broad category of AI, ML is a classical technique that provides algorithms that parse data, learn from data, and then apply what they have learned to make informed decisions. However, the limits of this technique are almost always related to the limits of the examples available. In addition to being correct, the latter should be complete, representing the physical problem to be analyzed in all its modalities. DL methods are a newer application of ML. The DL model usually employs the deep classification skills developed on another classification problem (pre-trained networks), and this acquired knowledge is "tuned" to the examples (usually limited in number) of the specific problem to be analyzed.

Although CDH disease is well suited to an integrated, ML- and DL-based analysis, the retrospective design of the study and the rarity of the disease could limit our findings. In addition, our results may not represent the disease’s intrinsic variability because of the limited sample size, and data collection might be incomplete or non-homogeneous. However, data will be mostly obtained from electronic medical records and computerized monitoring systems, thus mitigating the limits of a retrospective data collection.

A high-dimensional multivariate problem, with a large number of variables compared to the number of cases analyzed, could be hard to be solved through an ML approach due to a peculiar phenomenon known as the “curse of dimensionality” because the number of samples needed to estimate an arbitrary function with a high level of accuracy grows exponentially with the number of variables (i.e., dimensionality). The dimensionality reduction approaches listed in the “Analysis” section will contrast these issues.

In recent years, deep neural networks, particularly convolutional neural networks (CNN), have aroused great interest in the field of medical imaging [92]. This is undoubtedly due not only to the high classification performance demonstrated by these methods but also to the ease of carrying out a classification process using these tools. Indeed, the traditional chain consisting of preprocessing, feature extraction, and training models is entirely replaced by CNNs, including feature extraction in their training process. However, due to the limited number of training examples, a failure of the classification performance of the implemented ML methods could occur.

Despite being limited in size, which is intrinsically related to the nature of the disease, our cohort will have the advantage of presenting a broad spectrum of disease severity, including patients undergoing prenatal treatment and postnatal extracorporeal support. These patients represent a deep interest category due to the disease rarity and the limited number of Institutions performing FETO and neonatal ECMO. Therefore, the heterogeneity of our study population will represent a strength point for the study’s purposes.

## Ethics and dissemination

The present study will be in accordance with the principles of good clinical practice and the Helsinki Declaration. This study was approved by the local ethics committee (Milan Area 2, Italy) with approval number/ID 800_2020bis. However, due to the retrospective nature of the study, informed consent was waived by the Ethics Committee.

The study was registered at ClinicalTrials.gov with the identifier NCT04609163. Confidentiality of information will be guaranteed in accordance with the regulation in force.

Once the investigation is completed, we intend to publish our results in a peer-reviewed journal. Moreover, findings will be presented at relevant national and international conferences for fetal surgery, neonatology, pediatrics surgery, pediatric radiology, and computer science. Finally, the full database will be made free and available to share with the scientific community to further advance the knowledge in this field and promote collaborations (preserving medical confidentiality and in full respect for patients’ privacy).

Eventually, the ML predicting tools may be presented worldwide to CDH fetal and neonatal referral centers, with the ultimate goal to ease the management of patients and their family from the prenatal to the postnatal epoch.

## Conclusions

The predictive algorithms based on AI will provide an essential contribution for precise outcome prediction, early targeted interventions, and personalized management of fetuses and newborns with CDH. Furthermore, developing a ML- and DL-based prognostic pattern could enable a more rational resource allocation, non-invasive diagnostic evaluation, cost-effective and timely therapeutic management with a potential impact in terms of improved outcomes, reduced social burden, and economic savings. This will further accelerate the development of a precision medicine approach in the high-risk perinatal setting.

## Supporting information

S1 Table Data collection. Main prenatal, postnatal, and radiological parameters collected for ML-based analysis.
(DOCX)
