# Supplementary material for: A maChine and deep Learning Approach to predict pulmoNary hyperteNsIon in newbornS with congenital diaphragmatic Hernia (CLANNISH): Protocol for a retrospective study
Source: PLoS One. 2021 Nov 9;16(11):e0259724. doi: 10.1371/journal.pone.0259724 (PMC8577746; doi:10.1371/journal.pone.0259724)
Supplement: S1 Table — Main prenatal, postnatal, and radiological parameters collected for ML-based analysis. (DOCX) [file pone.0259724.s001.docx]

| **S1 Table. Main prenatal, postnatal, and radiological parameters collected for ML- and DL-based analysis.** | |
| --- | --- |
| **Prenatal Data** | |
| General maternal data | Age  Ethnicity  Previous gestations  Associated medical conditions |
| Pregnancy data | Mode of conception  Genetic testing (Amniocentesis/CVS, karyotyping, Array-CGH)  Premature rupture of membrane  RDS prophylaxis |
| Fetal US data  (25^+0^ - 30^+6^ weeks of gestation) | GA at diagnosis  Side of CDH  O/E LHR (tracing method) at diagnosis  Herniated liver/stomach/spleen  Position of the herniated stomach  Grading of hernia severity  Estimated fetal weight (EFW)  Amniotic fluid (AF)  Umbilical artery pulsatility index (UA PI)  Pulmonary artery pulsatility index (PI)  Pulmonary artery peak systolic velocity (PSV)  Pulmonary artery peak early diastolic reversed flow (PEDRF) |
| FETO procedure | Date of balloon insertion  GA at balloon insertion  Initial O/E LHR  Date of balloon removal  GA at balloon removal  Final O/E LHR  Mode of removal (endoscopic vs. EXIT) |
| **Postnatal Data** | |
| General neonatal data | GA at birth  Birthweight  Mode of delivery  Sex  APGAR score  Serial auxologic parameters (weight, length, cranial circumference)  Growth percentile  Death  Age at death  Length of stay |
| Surgical course | Day of surgery  Type of surgical repair  Prosthetic patch (diaphragmatic/abdominal)  Intra- or post-surgical complications  In-hospital recurrence |
| ECMO | Date of ECMO Start/Stop  Duration  Survival to ECMO |
| Cardiocirculatory | Heart rate  Arterial blood pressure (systolic, diastolic, mean)  vasoactive and inotropic support (type, dose, duration)  Pre- and postductal oxygen saturation  sPAP from tricuspid valve regurgitation  mPAP from pulmonary valve regurgitation  Pulmonary artery flow  PH grading (subsystemic, isosystemic, suprasystemic)  PDA  Presence and characteristics of shunts  Characteristics of intraventricular septum |
| Respiratory | Type and duration of mechanical ventilation  Oxygen supplementation (FiO_2_, duration)  Pulmonary vasodilators (type, dose, duration) |
| Hematologic | Blood components transfusions (type, number of transfusions)  Hemoglobin  Bilirubin |
| Infectious | Antibiotics (type, dose, duration)  Sepsis  C-reactive protein  Complete blood count and differential |
| Neurological | IVH or other CNS lesions |
| **Radiological Parameters** | |
| Fetal MRI  (25^+0^ – 35^+6^ weeks, before FETO) | GA at MRI  Fetal lung volume (total, right, left)  O/E TFLV  Liver volume  Percentage of liver herniation (%LH)  Mediastinal Shift Angle (MSA)  Apparent Diffusion Coefficient (ADC) |
| Chest X-ray  (Within 24 hours after birth) | Radiographic pulmonary area (total, ipsilateral, contralateral) |
| CDH: congenital diaphragmatic hernia; CNS: central nervous system; CVS: chorial villus sampling; DL: deep learning; ECMO: extracorporeal membrane oxygenation; EXIT: ex utero intrapartum treatment; FETO: fetal endoscopic tracheal occlusion; GA gestational age; IVH: intraventricular hemorrhage; ML: machine learning; mPAP: mean pulmonary artery pressure; MRI: magnetic resonance imaging; O/E LHR: observed/expected lung-to-head ratio; O/E TFLV: observed/expected total fetal lung volume; PDA: patent ductus arteriosus; PH: pulmonary hypertension; RDS: respiratory distress syndrome; sPAP: systolic pulmonary artery pressure. | |
